# Educational Data Mining Techniques for Student Performance Prediction: Method Review and Comparison Analysis

**DOI:** 10.3389/fpsyg.2021.698490

**Published:** 2021-12-07

**Authors:** Yupei Zhang, Yue Yun, Rui An, Jiaqi Cui, Huan Dai, Xuequn Shang

**Affiliations:** ^1^School of Computer Science, Northwestern Polytechnical University, Xi'an, China; ^2^Key Laboratory of Big Data Storage and Management, Ministry of Industry and Information Technology, Xi'an, China

**Keywords:** personalized education, review and discussion, educational data mining (EDM), student performance prediction, pattern recognition

## Abstract

Student performance prediction (SPP) aims to evaluate the grade that a student will reach before enrolling in a course or taking an exam. This prediction problem is a kernel task toward personalized education and has attracted increasing attention in the field of artificial intelligence and educational data mining (EDM). This paper provides a systematic review of the SPP study from the perspective of machine learning and data mining. This review partitions SPP into five stages, i.e., data collection, problem formalization, model, prediction, and application. To have an intuition on these involved methods, we conducted experiments on a data set from our institute and a public data set. Our educational dataset composed of 1,325 students, and 832 courses was collected from the information system, which represents a typical higher education in China. With the experimental results, discussions on current shortcomings and interesting future works are finally summarized from data collections to practices. This work provides developments and challenges in the study task of SPP and facilitates the progress of personalized education.

## 1. Introduction

Educational data mining (EDM), a very young research field, focuses on learning latent patterns in various educational situations, including student's knowledge analysis (Yeung and Yeung, 2018), student's learning behavior analysis (Juhaňák et al., 2019), teacher's curriculum planning (Reeves, 2018), course time arrangement (Zhang et al., 2018a). All involved studies have the final goal that is to improve the student learning performance (Liu et al., 2018, 2019; Anand, 2019; Wang et al., 2020), as well as other additional goals like reducing educational costs (Gronberg et al., 2004). As a result, in the past decades, various researches were concentrated on student performance prediction, referred to as SPP in this paper, (Sweeney et al., 2015; Polyzou and Karypis, 2016; Thanh-Nhan et al., 2016; Cakmak, 2017; Hu et al., 2017; Morsy and Karypis, 2017) or were evaluated by the student's final grades (Al-Radaideh et al., 2006; Shovon et al., 2012; Ahmed and Elaraby, 2014; Meier et al., 2015; Al-Barrak and Al-Razgan, 2016). While several review papers have summarized previous EDM research studies (Shahiri and Husain, 2015; Saa, 2016), this paper provides a more completed survey on the problem of SPP from the perspective of machine learning and data mining.

Student academic performance has various definitions varying from difficult points of view, but the quantified evaluation plays an important role in current educational institutions. SPP makes great sense to aid all stakeholders in the educational process. For students, SPP could help them choose suitable courses or exercises and make their plans for academic periods (Ibrahim and Rusli, 2007). For instructors, SPP can help adjust learning materials and teaching programs based on the student's ability and find the at-risk students (Bayer et al., 2012; Kloft et al., 2014). For educational managers, SPP could help to check the curriculum program and to optimize the course system (Reeves, 2018). Overall, stakeholders in the educational progress could have better plans to improve the education performance. Besides, the data-driven SPP study provides an objective reference for the education system.

Student performance prediction can be formulated into different problems in various situations. In this paper, we define the SPP problem in the general machine learning formulation, shown as follows:

**Problem 1 (SPP)**. Denote by $D=\left\{\left({\text{s}}_{1},{\text{c}}_{1},y_{1,1}\right),...,\left({\text{s}}_{n},{\text{c}}_{m},y_{n,m}\right)\right\}$ the educational data, where **s**_*i*_ presents the student-wise features, **c**_*j*_ presents the course-wise features, and *y*_*i, j*_ is the *i*-th student's grade on the *j*-th course. The goal of SPP is to seek a mapping M such that $M\left({\text{s}}_{i},{\text{c}}_{j}\right)=y_{i,j}$.

In Problem 1, student-wise features include student demographics that affect the course grade, while course-wise features include course descriptions that affect the course grade. In general, the grade is produced from the event that a student enrolls in a course, where all educational information is usually divided into student type and course type.

Based on the above problem definition, there are five general steps to solve Problem 1, i.e., data pre-processing and feature selection, problem reformulation, model learning, performance prediction, and result analysis. More specifically, the five steps could be shown as follows:

1. The first step is to collect data from the special SPP situation. As is shown in Problem 1, the data could consist of the triple {student, course, grade} to describe the scoring event. Student-wise features include age, sexual, healthy, economy, education level, etc. In contrast, course-wise features include frequent, duration, scale, open season, etc. (Elbadrawy et al., 2014; Kennedy et al., 2015; De Barba et al., 2016). The extended features for students could be parent's features, classmate-group's features, learning-records' features, etc and for courses could be instructor's features, prerequisite courses' features, assistants' features, etc. For the grade, there are three broadly used models: passed-failed model, grade model, and score model. Note that it is called grade for clarity in this paper. In addition, the learning situation could be divided into offline classrooms, online classrooms, and blending classrooms (Rovai and Jordan, 2004).
2. After the data is prepared, the second step aims to reformulate Problem 1. In general, Problem 1 is reformulated into clustering, classification, and regression. The clustering formulation is to group the $\text{X}={\left\{{\text{s}}_{i},{\text{c}}_{j}\right\}}_{i=1,j=1}^{n,m}$ into multi-clusters, where each cluster contains the instances with high similarities. Many studies partition **X** into different clusters based on students and/or courses in SPP (Cakmak, 2017). The classification formulation aims to predict the discrete grade using a machine-learning classifier, such as logic regression (Elbadrawy et al., 2014) and support vector machine (SVM) (Xu and Yang, 2016). The regression formulation is to predict the continued grade by using a regression model, such as linear regression (LR) (Alario-Hoyos et al., 2016) and neural networks (Oladokun et al., 2008). Besides, many studies transfer continuous scores into discrete grades (Shahiri and Husain, 2015).
3. In the third step, the chosen machine-learning model is developed to build the mappings M for the reformulated problem. Many studies employed the traditional machine-learning methods, such as decision trees (DTs) (Al-Radaideh et al., 2006; Koprinska et al., 2015), neighborhood method (Meier et al., 2015), LR (Anozie and Junker, 2006), neural networks (Andrews et al., 1995; Sorour et al., 2014), and kernel-based method (Boser et al., 1992). The new feature-learning techniques have been investigated in SPP, such as Lasso regression (Sorour et al., 2014; Zhang et al., 2018b; Zhang and Liu, 2020), matrix factorization (MF) (Slim et al., 2014), tensor factorization (TF) (Thai-Nghe et al., 2011a), and deep neural networks (Kim et al., 2018). In these methods, MF and deep learning have attracted increasing attention for SPP. However, simple methods can show more meaningful interpretations than a complex learning model (Van Merrienboer and Sweller, 2005).
4. With the learned model, the fourth step could predict the grade for a new student on a new course. That is, the new instance {**s**_*p*_, **c**_*q*_} is fed into M to achieve the *y*_*p, q*_. In this step, the current studies often have different strategies. The works (Al-Radaideh et al., 2006; Shovon et al., 2012; Ahmed and Elaraby, 2014; Meier et al., 2015; Al-Barrak and Al-Razgan, 2016) predicted a course grade of the student involved in training data, while the work (Ren et al., 2018) predicted the next-term grade based on the grade records. Besides, several studies predicted the course grade following the progress of a whole education period (Xu et al., 2017). However, few studies are focused on the pattern learning from Problem 1, ignoring the specific student or course.
5. When the model delivers results, it is hoped that the result could show some explainable patterns to help the stakeholders improve their respective tasks in education. In general, SPP provides explanations to the issues of students in route teaching and learning, e.g., the students at risk of dropout (Quadri and Kalyankar, 2010), the students of different knowledge statuses (Meier et al., 2015), the key factors to learning (Mayilvaganan and Kalpanadevi, 2014), and the course associations (Zhang et al., 2021a). A grading system that could predict the student grade in education progress might be a good tool to improve outcomes.

The remainder of this paper is organized as follows. Section 2 reviews the studies, including data collection, problem formulation, the used method, performance prediction, and practical application. Section 3 shows two evaluations using traditional machine learning methods on two data sets. Section 4 discusses current works and future problems, and Section 5 concludes this paper.

## 2. A Review on SPP Process

In this section, we summarizes the existing literature by a systematic review of SPP. As mentioned above, the existing studies in the five stages include (1) Data collection, literature pays attention to the tasks that are mainly dependent on the data information in hand. (2) Problem formulation, as mentioned in the literature, the research mainly consists of three formulations from their faced problems. (3) The used methods, various machine learning methods 2.2 are employed toward solving individual situations. (4) Performance prediction, the different evaluations are resulted from the used situations, e.g., the next-term prediction and the GPA prediction. (5) Practical application, the SPP models could be used under the complex real-world situations to aid students and teachers.

### 2.1. Data Collection

In the past decades, the study on SPP was mainly focused on the traditional classroom, where small datasets were collected in offline education. Now, online courses are being accepted by students and educational institutions, e.g., Coursera and edX, thus causing many kinds of research on the massive educational data from online education. Besides, the blending classroom that integrates both offline and online strategies provides a new path toward personalized education. Related literature is shown in Table 1, where many research focused on the online classroom due to MOOCs and data enrichment.

**Table 1:** The works studied in different situations.

**Data source**	**Sub-source**	**Reference**	**Count**
Offline classroom		Al-Radaideh et al., 2006; Nghe et al., 2007; Dekker et al., 2009; Bayer et al., 2012; Shovon et al., 2012; Ahmed and Elaraby, 2014; Elbadrawy et al., 2014; Mayilvaganan and Kalpanadevi, 2014; Sorour et al., 2014; Koprinska et al., 2015; Luo et al., 2015; Meier et al., 2015; Sweeney et al., 2015, 2016; Al-Barrak and Al-Razgan, 2016; Polyzou and Karypis, 2016; Thanh-Nhan et al., 2016; Hu et al., 2017; Morsy and Karypis, 2017; Ren et al., 2018	20
	Historical grade data & background information	Elbadrawy et al., 2014; Kennedy et al., 2015; Meier et al., 2015; De Barba et al., 2016; Lorenzen et al., 2017	5
Online classroom	Historical grade data & background information	Tabandeh and Sami, 2010; Thai-Nghe et al., 2010a,b, 2012; Toscher and Jahrer, 2010; Yu et al., 2010; Dietz-Uhler and Hurn, 2013; Goda et al., 2013; Elbadrawy et al., 2014, 2016; Kloft et al., 2014; Sorour et al., 2014; Hwang and Su, 2015; Thai-Nghe and Schmidt-Thieme, 2015; Xu and Yang, 2016; Adejo and Connolly, 2017; Yang et al., 2017; Su et al., 2018	18
	Historical grade data & background information	Kloft et al., 2014; Wen et al., 2014a,b; Arguello and Shaffer, 2015; Koprinska et al., 2015; Wang et al., 2015; Wong et al., 2015; Lu et al., 2017; Gitinabard et al., 2018	9
Blending classroom		Elbadrawy et al., 2014; Sorour et al., 2014; Koprinska et al., 2015; Meier et al., 2015; Zacharis, 2016	7

#### 2.1.1. Offline Classroom

In traditional education, students usually finish academic courses in offline classrooms, where the research data could be obtained. The data from the offline classroom is usually composed of student learning records, courses, and teachers. On the obtained data, various grade prediction methods are employed to conduct data analysis, which can be grouped into statistical methods and pattern recognition methods, such as (Al-Radaideh et al., 2006; Dekker et al., 2009; Shovon et al., 2012; Ahmed and Elaraby, 2014; Meier et al., 2015; Sweeney et al., 2015; Al-Barrak and Al-Razgan, 2016; Polyzou and Karypis, 2016; Thanh-Nhan et al., 2016; Morsy and Karypis, 2017). To pursuit a better performance on SPP, the societal background information is considered in terms of various metrics (Nghe et al., 2007; Elbadrawy et al., 2014; Mayilvaganan and Kalpanadevi, 2014; Koprinska et al., 2015; Sweeney et al., 2016; Thanh-Nhan et al., 2016; Hu et al., 2017; Ren et al., 2018). The background data usually contains student demographics (parents' education, family income, household registration), curriculum plans, teachers' quality and style, and student performance evaluation. Specially, the work of Hu et al. showed that background features significantly improved the prediction model performance (Hu et al., 2017).

In addition to these above attributions, behavior features were also considered, e.g., the social dependence relationship obtained from emails and social networks (Bayer et al., 2012). This study combined social behavior features with background attributions to train the prediction model and finally achieved an improvement the prediction accuracy by 10%. The comments from students after each lesson were considered by Sorour et al. (2014) and Luo et al. (2015). These comments show student learning attitude, subject understanding, course difficulty, and activity in a classroom. Especially, Koprinska et al. (2015) explored multiple data sources, including demographics, social behaviors, and academic data. Their experiments analyzed the most important features and then discussed how to use them to improve teaching and learning.

#### 2.1.2. Online Classroom

Recently, with the development of online learning platforms, e.g., MOOCs (Massive Open Online Courses), students choose to learn the online courses or as supplements to a traditional classroom. More researchers paid an amount of attention to the online classrooms for their widely used range and massive educational records. The data could be easily obtained in an online classroom, as each educational activity is recorded with log files, e.g., click stream of the mouse, texts from discussions, learning-time length, etc.

**Historical performance data and background information**. Many studies on SPP in online classroom used historical performance data (Meier et al., 2015; Lorenzen et al., 2017), students' background information, course's descriptors, and teachers' background (Elbadrawy et al., 2014; Kennedy et al., 2015; De Barba et al., 2016) to train their prediction models. Kennedy et al. analyzed the grade information, course background information, and an event log of interactions from 6,635 learners (Kennedy et al., 2015). Then, Kennedy found that prior knowledge is the most significant predictor of MOOC success, followed by students' ability to revisit their previous work.

**Data from the log file**. The log file is an important characteristic that distinguishes online classrooms from traditional offline classrooms, where the log file could easily record the online-learning-process data. Many researchers explored these processing data to predict student grades, e.g., (Thai-Nghe et al., 2010b; Toscher and Jahrer, 2010; Elbadrawy et al., 2014). For instance, Su et al. used both exercise records and the question texts to model the student exercising process (Su et al., 2018); researchers made attempts to understand the performance of individual students deeply by analyzing the comments from students. Student's comments can reflect their learning attitudes to the lesson, understanding of subjects, difficulties to learn, and learning activities, which potentially associate to the grade (Dietz-Uhler and Hurn, 2013; Goda et al., 2013; Sorour et al., 2014). In these data sets, the educational data set from the Knowledge Discovery and Data Mining Cup is widely used for validation, i.e., the process records from learning the course of "Algebra" and learning the course of "Bridge To Algebra" (Tabandeh and Sami, 2010; Thai-Nghe et al., 2010a, 2012; Toscher and Jahrer, 2010; Yu et al., 2010; Hwang and Su, 2015; Thai-Nghe and Schmidt-Thieme, 2015). The two data sets take the form of interaction records between students and computer-aided-tutoring systems. Students solve problems in the tutor system, and each interaction between student and system was logged as a transaction. Four key terms form the building blocks of our data, i.e., problem, step, knowledge component, and opportunity and step start time, first transaction time, correct transaction time, and so on. Especially, before training the prediction models, (Thai-Nghe et al., 2012) selected the features that are more related to student performance.

In addition, the click-stream was recorded in the log file. The click-stream data includes thousands of weblog records which can be generally classified into two types (Kloft et al., 2014; Xu and Yang, 2016; Yang et al., 2017): (1) the page view log, including the number of requests, the number of active days, the number of page views, the number of homework page views, and so on. (2) lecture video log, including the number of requests, the number of video views, the number of start-stop during video plays, the number of re-listening during video views, and so on. Many researchers attempted to apply these data of click-stream to model the state of learning of students and training the prediction model (Elbadrawy et al., 2014, 2016; Xu and Yang, 2016; Adejo and Connolly, 2017).

**Data from the discussion forum**. The discussion forum is another important characteristic that distinguishes online classrooms from the traditional offline classroom. All the students could discuss the course or the problems with each other (Kloft et al., 2014; Wen et al., 2014a; Arguello and Shaffer, 2015; Wong et al., 2015). In this way, researchers obtained massive behavior records to train the prediction models. The forum data is broadly obtained from the student's posts. Participants usually create a thread by making a root post and reply to existing threads by adding comments at the end. Several papers predicted student performance using the student's active forum data including submissions, numbers of forum posts, length of the forum thread, and so on (Wen et al., 2014b; Koprinska et al., 2015; Wang et al., 2015; Lu et al., 2017; Gitinabard et al., 2018). Furthermore, click stream and forum data are also integrated to enhance the grade predication (Koprinska et al., 2015; Lu et al., 2017; Gitinabard et al., 2018). (Gitinabard et al., 2018) applied a combination of modeling and feature-selection methods to identify the important features in both dropout and certification prediction. The author analyzed the discussion texts to obtain the social relationships of a learner, e.g., two posts that shared the same root thread. In this research, the author also considered the forum features, including forum activities and the submission counts.

#### 2.1.3. Blending Classroom

In the blending context of the offline and online classrooms, researchers obtain more attributions for student performance from multiple data sources and obtain better prediction performance (Rovai and Jordan, 2004; Elbadrawy et al., 2014; Sorour et al., 2014; Koprinska et al., 2015; Meier et al., 2015; Zacharis, 2016). Nick et al. used student data stored in MOOCs. They predicted student success based on four learning activities: communication *via* emails, collaborative content creation with wiki, content interaction measured by files viewed, and self-evaluation through online quizzes. Next, a model based on the Multi-Layer Perceptron Neural Network was trained to predict student performance in a blended learning course environment. The model predicted the performance of students with a correct classification rate of 98.3% (Zacharis, 2016).

### 2.2. Problem Formulation

In EDM methods, predicting student learning performance is a problem that maps student information to his/her grades. Usually, this problem could be formalized into machine learning problems, i.e., clustering, classification, and regression. Here, we generally give the formulations of prediction models in the study of SPP. Denote the training dataset by $D=\left\{\left({\text{s}}_{i},{\text{c}}_{j},y_{i,j}\right)|i=1...n,j=1...m\right\}$, where *y*_*i, j*_ is the grade of *i*-th student that obtained on *j*-th course, *n* and *m* are the number of students and courses, respectively.

1. **Clustering**. The works of Oyelade et al. (2010) and Hwang and Su (2015) formalized SPP into a cluster problem, where the students are grouped into multi-clusters **G** = {**g**_1_...**g**_*k*_}, where *k* is the number of clusters and then the objective student's performance is predicted in the specific cluster. The problem could be defined as follows:
  **Problem 2 (SPP-Clustering)**. The goal of SPP-Clustering is to seek a mapping of clustering $M_{1}$ such that $M_{1}\left(\text{S},\text{C}\right)=\text{G}$. To predict an object student that lies in the cluster **g**_*k*_, a new mapping $M_{2}$ is built such that $M_{2}\left({\text{s}}_{i},{\text{c}}_{j}\right)=y_{i,j}$ for (**s**_*i*_, **c**_*j*_) ∈ **g**_*k*_.
  Usually, $M_{2}$ is created by computing the average value of the performance of all students of **g**_*k*_ on course **c**_*j*_ (Cakmak, 2017).
2. **Classification**. When researchers consider the SPP task as a classification task, the prediction output is the discrete grades for a student, e.g., GPAs (Veloski et al., 2000), pass/fail (Thai-Nghe et al., 2009; Bayer et al., 2012; Abu-Oda and El-Halees, 2015), or others (Elbadrawy et al., 2014; Hu et al., 2017; Morsy and Karypis, 2017). Let **Y** = {**y**_*i, j*_} be the label set of different classifications, and the value of **y**_*i, j*_ is one element of the label set, ℓ_1_,..., ℓ_*k*_.
  **Problem 3 (SPP-Classification)**. The goal of SPP-Classification is to seek a mapping $M_{3}$, such that $M_{3}\left({\text{s}}_{i},{\text{c}}_{j},\text{A}\right)=max\left\{{\text{p}}_{1},...,{\text{p}}_{k}\right\}=y_{i,j}$, where *p*_*k*_ is the possibility of (**s**_*i*_, **c**_*j*_) belonging to ℓ_*k*_.
  Here, the student's grades are generally divided into several categories, like A, B, C, and D (Xu et al., 2017), according to their scores.
3. **Regression**. The regression model is a function that represents the mapping between input variables and output variables. The regression problem is equivalent to function fitting: selecting a function curve to fit the known data well and predict the unknown data well. In SPP, regression techniques are often used to predict the continuous scores of students in specific courses (Polyzou and Karypis, 2016; Hu et al., 2017; Morsy and Karypis, 2017).
  **Problem 4 (SPP-Regression)**. The goal of SPP-Regression is to seek a mapping $M_{4}$, such that $M_{4}\left({\text{s}}_{i},{\text{c}}_{j},\text{A}\right)=y_{i,j}$, where *y*_*i, j*_ is usually continuous scores.

### 2.3. Current Methods

As mentioned above, there are mainly three problem formulations in SPP. Here, we make a systematic review of the methods used in SPP, as follows. Table 2 shows the statistic of those related researches.

**Table 2:** The related works with different machine learning models.

**Proposed methods**	**Problem formulation**	**Reference**	**Count**
Decision trees	Classification	Safavian and Landgrebe, 1991; Al-Radaideh et al., 2006; Nghe et al., 2007; Dekker et al., 2009; Thai-Nghe et al., 2009; Bunkar et al., 2012; Shovon et al., 2012; Koprinska et al., 2015; Al-Barrak and Al-Razgan, 2016; Saa, 2016	10
Linear regression	Regression	Tabandeh and Sami, 2010; Elbadrawy et al., 2014; Kennedy et al., 2015; Meier et al., 2015; Wang et al., 2015; Alario-Hoyos et al., 2016; De Barba et al., 2016; Polyzou and Karypis, 2016; Ren et al., 2016; Hu et al., 2017; Lorenzen et al., 2017; Morsy and Karypis, 2017	13
Support vector machines	Classification	Kentli and Sahin, 2011; Bydžovská, 2016; Xu and Yang, 2016	4
Matrix factorization	Regression / Clustering	Lee and Seung, 2001; Thai-Nghe et al., 2010a, 2011a, 2012; Toscher and Jahrer, 2010; Bokde et al., 2015; Hwang and Su, 2015; Sweeney et al., 2015; Thai-Nghe and Schmidt-Thieme, 2015; Elbadrawy et al., 2016; Polyzou and Karypis, 2016; Hu et al., 2017; Lorenzen et al., 2017; Ren et al., 2018; Zhang et al., 2020c	15
Collaborative filtering	Classification / Clustering	Sheena et al., 2000; Schafer et al., 2007; Li and Zaman, 2014; Bydžovská, 2015; Meier et al., 2015; Cakmak, 2017; Jyoti and Walia, 2017	7
Artificial neural network	Classification / Clustering / Regression	Andrews et al., 1995; Oladokun et al., 2008; Sorour et al., 2014; Luo et al., 2015; Shahiri and Husain, 2015; Młynarska et al., 2016; Zacharis, 2016; Yang et al., 2017; Su et al., 2018	9
Deep learning	Classification / Clustering / Regression	Guo et al., 2015; Yang et al., 2017; Kim et al., 2018; Hu and Rangwala, 2019a,b	5
Other methods	Regression / Clustering	Slim et al., 2014; Li et al., 2016 Iqbal et al., 2017	3

#### 2.3.1. Decision Trees

Decision trees (DTs) are a non-parametric supervised learning method used for classification and regression. It learns the splitting rule to divide the data according to their features and obtains the labels by voting at leaf nodes (Safavian and Landgrebe, 1991).

Decision trees could deliver interpretable results and thus obtain much attention for SPP (Al-Radaideh et al., 2006; Nghe et al., 2007; Dekker et al., 2009; Thai-Nghe et al., 2009; Bunkar et al., 2012; Shovon et al., 2012; Koprinska et al., 2015; Al-Barrak and Al-Razgan, 2016; Saa, 2016). The tree model can be transformed into a set of "if-then" rules that are intuitive and easy to understand by human beings. Al-Barrak et al. studied and evaluated the "if-then" rules to improve prediction accuracy in the higher education system (Al-Barrak and Al-Razgan, 2016). Based on different feature selection methods and pruning rules, the DT model has three main algorithms, i.e., ID3, CART, and C4.5. Bunkar et al. compared the three DT algorithms. They carried out experiments to seek the best one (Bunkar et al., 2012). Among these DT algorithms and other machine learning algorithms, the DT showed a higher precision on their used data set. Nghe et al. investigated the decision tree and the Bayesian Network to predict the academic performance of undergraduates and postgraduates from two academic institutions. In their experiment, the accuracy of the DT is always 3–12% higher than the Bayesian Network (Nghe et al., 2007).

Many studies used the ensemble algorithm to combine the DT with other models (Dekker et al., 2009; Thai-Nghe et al., 2009; Bunkar et al., 2012; Shovon et al., 2012). For example, when predicting pass or fail in an exam, the main problem is that the number of "passed" students is much higher than the number of "failed" students. The prediction results are dropped down due to this imbalance issue. Thai-Nghe et al. proposed to address the problem of class imbalance through over-sampling techniques and used the cost-sensitive learning (CSL) method to improve the prediction (Thai-Nghe et al., 2009). The authors first re-balanced data sets then used the DT on the balanced data. Compared with the original data set, the results were significantly improved. There is also an imbalance problem in predicting student dropout (Dekker et al., 2009). The DT and CSL are combined in the experiments to predict student dropout, where the decision tree gives an accepted accuracy of about 80%.

In online classrooms, researchers also integrated multiple data sources to improve the performance of the DT. Koprinska et al. considered multiple data sources, including click-streams, submission steps of an academic task and outcomes in an automatic marking system, assessment marks in a semester, and student engagement with discussion forums, to build an improved DT classifier (Koprinska et al., 2015). The results showed that multiple data sources could improve the prediction accuracy compared to the single data source.

#### 2.3.2. Linear Regression

In statistics, LR is a linear approach to modeling the relationship between a scale response and one or more explanatory variables. The case of one explanatory variable is referred to as one variable LR, while for more than one explanatory variable, it is referred to as multi-variable LR.

A way to model the problem of grade prediction is to take into account the academic degree program. Degree program always requires students to take a set of courses in order, due to the knowledge provided by the previous courses being essential for subsequent courses (Tabandeh and Sami, 2010; Wang et al., 2015; Alario-Hoyos et al., 2016; Ren et al., 2016; Morsy and Karypis, 2017). With this idea, Polyzou et al. developed course-specific regression (CSR) (Polyzou and Karypis, 2016), and predict student grades in a course using a sparse linear combination (Zhang and Liu, 2020). Following this way, there are many improved works (Polyzou and Karypis, 2016; Hu et al., 2017; Morsy and Karypis, 2017). While the CSR model fails to consider the side-factors for student performance, Hu et al. proposed to combine content features with CSR models (Hu et al., 2017). They extracted features related to students and courses and incorporated these features into the prediction model. However, the LR model suffers from the sparsity problem when there are many elective courses. To address this limitation, Polyzou et al. developed a sparse LR method for student-specific regression (SSR), using the student-course-specific grade matrix. Elbadrawy et al. (2014) proposed to enhance the student-specific grade models in the LR model to predict student performance. Thus, each student was predicted by a specific LR model. Its advantage is that the models could exploit all student's historical grades and thus mitigate the data sparsity issue. On their used data, the multiple LR models achieve an RMSE of 0.147, while the traditional single LR model obtained an RMSE of 0.177, benefiting from the consideration of individual information.

#### 2.3.3. Support Vector Machines

In machine learning, SVMs are very effective supervised learning models that could be used for both classification and regression. SVM splits the data by seeking the maximized margin between two classes (Cortes and Vapnik, 1995). Due to SVM's powerful capability of classification, it has been investigated many times for SPP studies or used as a baseline method.

According to psychology, the behaviors potentially affect the student evaluation. Xu et al. divided students into three categories based on the detailed records of learning activities on MOOCs platforms, i.e., certification earning, video watching, and course sampling (Xu and Yang, 2016). Then, the authors built a predictor based on SVM to predict certification obtaining (Cortes and Vapnik, 1995). Fulya et al. (Kentli and Sahin, 2011) employed SVM on 504 data records from the classroom to predict the GPA. Hana et al. (Bydžovská, 2016) compared the traditional machine learning algorithms for SPP, including SVM, LR, Random Forest et al., where SVM is the best on both study-related data and social behavior data. However, SVM suffers from computation cost in big data due to its optimization limitation.

#### 2.3.4. Matrix Factorization

Matrix Factorization aims to decompose a matrix into two matrices, finding latent features between the two matrices (Bokde et al., 2015). For SPP, each element could be generalized by the product of a student representation and a course representation, where both representations are yielded in the latent feature space (Zhang et al., 2020c). That is, letting the student vector be the row of the raw matrix and the course vector be the column of the raw matrix, matrix competition aims to seek two latent feature metrics for student and course to approximate the original matrix student performance matrix (Thai-Nghe et al., 2010a, 2012; Toscher and Jahrer, 2010; Hwang and Su, 2015; Thai-Nghe and Schmidt-Thieme, 2015; Elbadrawy et al., 2016). However, except for the historical grades, there are many additional factors that influence student performance, such as the course difficulty, the quality and teaching style of the instructor, the academic level of students. Hu et al. proposed a hybrid LR-MF model that considered those features of students, courses, and instructors, to improve the performance of the curriculum-specific model (Hu et al., 2017). Ren et al. (2018) proposed additive latent effect models by incorporating the above factors to predict the student's next-term grades. The experimental results demonstrated that their methods significantly outperformed the baselines for SPP.

In the context of predicting the score of an exercise, MF was employed to implicitly encode "slip rate" (the probability that the student knows how to solve a question but makes a mistake) and the "guess rate" (the probability that the student does not know how to solve a question but guesses correctly) of the student in an examination, resulting in an excellent performance on the educational data set of Knowledge Discovery and Data Mining Cup 2010 (Thai-Nghe et al., 2010a). In (Lee and Seung, 2001; Hwang and Su, 2015), Non-negative MF (NMF) was used to integrate the non-negativity of student grades. TF was exploited to take temporal effects into account in the MF model (Thai-Nghe et al., 2011a), resulting from the improvements of the student's ability. Since grade matrix is an implicitly low rank, low-rank MF (LRMF) was investigated in the data sets from the online learning platform in the work of Lorenzen et al. (2017).

The MF-based model assumes a low-dimensional latent feature space that could represent both students and courses. However, the set of courses is usually an incomplete subset because courses are usually selected for various requirements. To address this problem, Polyzou et al. developed a course-specific MF (CSMF) method that estimates an MF model for each course, where a dense subset of the data could be available (Polyzou and Karypis, 2016). The dense course-specific matrix could make a more reliable estimation. In addition, the cold start is a necessary problem.... needed to be considered in SPP. Sweeney et al. proposed to combine Factorization Machines (FM) and Random Forests (RF) to create a hybrid model, taking advantage of both models to solve the cold-start problem (Sweeney et al., 2015).

#### 2.3.5. Collaborative Filtering

Collaborative filtering predicates a user's interests by seeking similar preferences from other users (Schafer et al., 2007). The underlying assumption is that if person A has the same opinions as person B on an issue, A is more likely to have B's opinion on another issue than a randomly chosen person. For instance, a CF recommendation system could predict which television show a user would like, given a partial subset of those user's tastes (likes or dislikes) (Sheena et al., 2000; Li and Zaman, 2014).

When applied to SPP, CF finds the most similar students with target students based on grade records. Sirikayon et al. performed various methods to calculate student similarity, including Pearson correlation, cosine similarity, and Euclidean distance. (Bydžovská, 2015). Their experiments showed that Pearson correlation achieves the lowest prediction error, where a prior course clustering could enhance predictability. Cakmak et al.'s work enhanced the standard CF by integrating automated outlier eliminations and GPA-based similarity filtering (Cakmak, 2017). Their methods estimated student course grades with an average error rate of 0.26, with an error improvement of 16%, compared with other methods.

In CF, the k-nearest neighbor algorithm (k-NN) is a non-parametric method used for classification, where prediction is based on the k nearest neighbors in given data (Jyoti and Walia, 2017). To predict the normalized score or grade of student **s** in performance *k* of year *y*, researchers often define the similarity or employ a traditional similarity metric. The performance of a student is then predicted on his/her k nearest neighbors. (Meier et al., 2015) derived a confident estimate on grade prediction and demonstrated the performance of the proposed algorithm on a real data set composed of 700 undergraduate students enrolled in the course of digital signal processing at UCLA in the past 7 years.

#### 2.3.6. Artificial Neural Network

Neural networks are a series of algorithms that endeavor to recognize underlying relationships in a set of data by mimicking the information process of the human brain. In this sense, neural networks refer to systems of neurons, either organic or artificial. ANN is a model composed of multiple neural layers and is trained in iterative optimization. ANN has a wide range of applications due to its power in modeling the approximation from inputs to outputs (Andrews et al., 1995). Hence, many studies used ANN to predict student performance (Oladokun et al., 2008; Sorour et al., 2014; Luo et al., 2015; Shahiri and Husain, 2015; Młynarska et al., 2016; Zacharis, 2016; Yang et al., 2017; Su et al., 2018). For instance, Oladokun et al. employed the multi-layer perception on the pre-admission data of five different university graduates for SPP, achieving an accuracy of about 74% (Oladokun et al., 2008).

Many researchers collected these features from student' self assesses by using ANN models to predict student's performance. Researchers asked for student' comments per lesson to reflect their learning attitude and understanding degree of course content and learning difficulty. With this data, Sorour et al. conducted experiments with the Latent semantic analysis (LSA) technique and ANN model (Sorour et al., 2014), achieving an average prediction accuracy of about 82.6%. Luo et al. employed Word2Vec and ANN to predict student grades in each lesson based on their comments (Luo et al., 2015). The experiment results showed that the prediction rate reached 80% on the 6 consecutive lessons, and a final prediction rate reached 94% from all 15 lessons. Tsung-Yen et al. trained a time series neural network based on both previous performance and click-stream data (Yang et al., 2017). The prediction model outperformed the method of using average past grades by more than 60%, and the lasso regression by more than 15%. To take all advantage of both students exercise records and the texts of exercises, Su *et al*. developed a novel Exercise-Enhanced Recurrent Neural Network (EERNN), where authors adopted a bidirectional LSTM to learn exercise representation from texts and then proposed the EERNN to trace student states in their sequential exercising process (Su et al., 2018).

#### 2.3.7. Deep Learning

The deep learning-based model, one of the powerful mapping-based methods, aims to learn deep nonlinear features from original features for sequent tasks and has become benchmarks in a wide range of applications in recent years (LeCun et al., 2015). To predict student performance, Guo et al. trained a student performance prediction network of six fully connected layers on the high-school data composed of background data, school-life data, past-study data, and personal descriptions (Guo et al., 2015). Yang et al. used a time series deep neural network to predict the evolution of a student's grade in massive open online courses (MOOCs) based on the data on student behaviors (Yang et al., 2017). Kim et al. (2018) recast the student performance prediction as a sequential event prediction and proposed a deep model, GritNet, for this problem by integrating the bidirectional long short-term memory (LSTM). To capture the sequential features of students grades in prior courses, Hu et al. modeled the learning behavior and performance using RNNs with LSTM for the next-course grade prediction (Hu and Rangwala, 2019b). Waheed et al. showed that deep neural networks achieved a higher prediction accuracy than logistic regression and SVMs on the clickstream data (Yang et al., 2017). Hu et al. proposed attention-based graph convolutional networks to predict next-term course grades based on past grades (Hu and Rangwala, 2019a). Yupei et al. proposed a sparse attention convolutional neural networks (SACNN) to predict undergraduate grades in Chinese higher education, where they not only achieve a good prediction accuracy but also gave the explanation for the question "why a student is predicted to pass/fail based on the course's association?" (Zhang et al., 2021a).

#### 2.3.8. Other Methods

Restricted Boltzmann Machine (RBM), an unsupervised machine learning technique, creates a bipartite graph composed of two network layers. The first layer is called the visible layer, which is used to receive data features. These nodes are connected to the second layer, called the hidden layer containing symmetrically weighted connections. Iqbal et al. investigated CF, MF, and RBM methods to predict student academic performance in the Information Technology University (ITU) (Iqbal et al., 2017). RBM technology was better than other techniques among all the mentioned methods on their data sets from the results.

Markov Network was also developed to predict the next-time course in a sequence (Slim et al., 2014). Slim et al. used MN to represent the curriculum graphs of a particular degree course. Based on GPA in a given semester, the MN model could predict GPA in the next semesters. They analyzed 400 students from the University of New Mexico (UNM) who have completed their degree programs. The mean square error (MSE) is used to measure the performance of the framework. The results showed that as the number of semester grades increases, MSE gradually declines (Slim et al., 2014).

In Li et al. (2016), the fuzzy-clustering model and multi-variable regression were combined into an framework to predict student academic performance. The authors considered both the historical scores and the attributes that are related to normal study behavior. In this study, students were clustered by using the fuzzy C-means model based on their existing academic records to discover the relationship between the required grade and the previous grades. By considering student behaviors, the similarity between the objective student and other students with similar academic records was calculated to generate an offset value. Finally, based on the cluster membership, the similarity, and the offset value, the objective grade was predicted in terms of a predefined linear system.

### 2.4. Performance Evaluation

With different goals, these studies of SPP focus on three-time spans of courses, i.e., single course grade prediction, the next-term performance prediction, and the whole learning period prediction. In this section, we reviewed the existing literature on SPP from their periods in SPP. The summary of these research studies is shown in Table 3.

**Table 3:** The list of references for performance evaluation.

**Performance evaluation**	**Reference**	**Count**
Single course grade prediction	Tabandeh and Sami, 2010; Thai-Nghe et al., 2010a, 2012; Toscher and Jahrer, 2010; Yu et al., 2010; Hwang and Su, 2015; Thai-Nghe and Schmidt-Thieme, 2015; Xu and Yang, 2016; Yang et al., 2017	9
The next-term performance prediction	Sweeney et al., 2015; Elbadrawy et al., 2016; Morsy and Karypis, 2017; Ren et al., 2018	4
Whole learning period's performance prediction	Oladokun et al., 2008; Vitulić and Zupančič, 2013; Meier et al., 2015; Al-Barrak and Al-Razgan, 2016; Hunt et al., 2017; Xu et al., 2017; Tampakas et al., 2018	7

#### 2.4.1. Single Course Grade Prediction

Many researchers focused on the single target course. They analyzed the score that the student would reach in the final exam or mid-term test (Tabandeh and Sami, 2010; Thai-Nghe et al., 2010a; Toscher and Jahrer, 2010; Yu et al., 2010). In these studies, most of the researchers are interested in the knowledge level of students on the target course and focused on one specific examination. The authors predicted the student's knowledge level and the student's grade using test questions (Thai-Nghe et al., 2012; Hwang and Su, 2015; Thai-Nghe and Schmidt-Thieme, 2015) and the process features of interest (Xu and Yang, 2016; Yang et al., 2017). Chein-Shung et al. developed a novel regularization framework that imposes locality preserving constraints into the weighted regularized nonnegative MF for SPP (Xu and Yang, 2016). The author predicted the performance on *Algebra* and *Bridge* by using exam question texts, solution steps, and skills. Tsung-Yen et al. incorporated richer data from the learning process of video watching, to train a time-series neural network, followed by predicting CFA scores of students.

#### 2.4.2. Next-Term Performance Prediction

Many researchers were focused on predicting student next-term performance to adjust the teaching plan (Sweeney et al., 2015; Elbadrawy et al., 2016; Morsy and Karypis, 2017; Ren et al., 2018). This research aims to estimate student learning performance on courses that are expected to engage in the next term. Students can use estimated grades to select courses for which they will perform well, thereby allowing them to make progress toward graduation. The estimated grade could also provide suggestions for the difficulty rating for courses, which helps students prioritize their studies and manage time schedules. Besides, course instructors and departments could also benefit from knowing student' registration on all courses. This enables them to make adjustments, such as holding additional office hours and allocating teaching assistants. Zhiyun et al. proposed additive latent effect (ALE) models that incorporate additive effects associated with students and courses to solve the next-term performance prediction. Especially, authors were able to highlight the improved prediction performance of ALE with the use of latent factors of course instructors, student academic levels, and student global latent effects (Ren et al., 2018).

#### 2.4.3. Performance Prediction in Entire Learning Period

The entire learning-period prediction predicts the indicators of the entire learning process, such as the GPA. This study aims to improve student's final GPA and graduation ratio (Oladokun et al., 2008; Meier et al., 2015). In these studies, researchers are mostly focused on the final GPAs (Vitulić and Zupančič, 2013; Al-Barrak and Al-Razgan, 2016) to trace student performance over the academic semesters (Hunt et al., 2017; Xu et al., 2017; Tampakas et al., 2018). Especially, they collected the performance of the student in each term of the whole learning period and other side information, e.g., background information of students/courses. Michael and Muna employed the J48 algorithm to predict the student's final GPA based on their transcripts and their course grades (Al-Barrak and Al-Razgan, 2016). Jie et al. proposed an ensemble method to predict students' future performance in degree programs, using the data of their current and past grades. A latent factor model-based course clustering method was developed to discover relevant courses as base predictors. An ensemble-based progressive prediction architecture was also developed to incorporate students' ability improvements into the prediction model. Additionally, this work could provide good suggestions on curriculum designs in degree programs (Xu et al., 2017).

### 2.5. Practical Application

As mentioned above, researchers studied the task of SPP with different goals and used the prediction results in different situations. Here, we listed some practical applications, shown in Table 4.

**Table 4:** The list of references of the practical application of SPP.

**Practical application**	**Reference**	**Count**
The recommendation system	Ray and Sharma, 2011; Thai-Nghe et al., 2011b; Denley, 2013; Elbadrawy and Karypis, 2016; Lian et al., 2016; Su et al., 2018	6
Early warning system	Blanchfield, 1971; Dekker et al., 2009; Quadri and Kalyankar, 2010; Bayer et al., 2012; Yang et al., 2013; Kloft et al., 2014; Abu-Oda and El-Halees, 2015; Lu et al., 2017; Gitinabard et al., 2018	9
Other applications	Jussim, 1989; Trouilloud et al., 2002; Hu and Huang, 2018; Lam et al., 2018; Juhaňák et al., 2019; Kushwaha et al., 2019; Supianto et al., 2019	7

#### 2.5.1. Recommendation System

Many researchers combined SPP tasks with recommendation systems to enhance education outcomes by making a personalized educational plan. To offer personalized exercise recommendations, Nguyen et al. proposed to use context-aware models for SPP by utilizing all interactions of the given student-task pairs. This approach could be applied in a personalized learning environment, such as recommending exercises to students and predicting student performance (Thai-Nghe et al., 2011b). Yu et al. proposed an EERNN framework for SPP by taking both students' exercise records to exercise texts into account. In EERNN, authors first designed a bidirectional LSTM to learn exercise representations from texts and then proposed a new network architecture to trace student states (i.e., knowledge states) in their sequential exercising process with the combination of exercise representations. To make final predictions, the authors designed two strategies under EERNN, i.e., EERNNM with Markov property and EERNNA with an Attention mechanism (Su et al., 2018). For library book recommendations, Defu et. al. proposed a supervised content-aware MF for mutual reinforcement of academic performance prediction based on library data (Lian et al., 2016). For course recommendation (Ray and Sharma, 2011; Denley, 2013; Elbadrawy and Karypis, 2016), Asmaa et. al. investigated how students and course academic features influence the enrollment patterns and then applied these key features to define student and course groups at various levels of granularity. Finally, the authors combined these groups with existing grade predictions and top-n course ranking models, e.g., neighborhood-based user collaborative filtering, MF, and popularity-based ranking approaches (Elbadrawy and Karypis, 2016).

#### 2.5.2. Early Warning System

The early warning system is a key application based on the study of SPP. In the context of traditional offline education, instructors wish to know the students who are at risk of dropping out or the students who possibly fail in the examination (Blanchfield, 1971; Dekker et al., 2009; Quadri and Kalyankar, 2010; Bayer et al., 2012; Abu-Oda and El-Halees, 2015). The online classroom is the same, where instructors wish to know the students under the risk or have a lower motivation to finish his/her tasks (Yang et al., 2013; Kloft et al., 2014; Lu et al., 2017; Gitinabard et al., 2018).

As mentioned above, many researchers study the common problem of dropping out. Bayer et al. studied the structured data of students' social behaviors, e.g., e-mail and discussion board conversations. They introduced learning a classifier for student failure prediction using a CSL method (Bayer et al., 2012). Especially, the authors described extraction features from both student data and behavior graph data. Niki et al. conducted a survival analysis to identify dropouts by a combination of modeling and feature selection methods. The author evaluated three different models under different definitions of dropout. Besides, the author assessed models over time by evaluating whether models learned on week 1 could predict dropouts in week 2 (Gitinabard et al., 2018).

#### 2.5.3. Other Applications

There are many other applications of SPP due to lots of studies using SPP as an evaluation. David et al. explored the relationships between teacher expectations and student achievements in physical education classes. Student achievement may confirm teacher expectations because these expectations create self-fulfilling prophecies, perceptual biases, and accurate predictions (Jussim, 1989). Another purpose was to examine the mediating role played by students' perceived ability in the teacher expectancy process (Trouilloud et al., 2002). Libor et al. studied the task of SPP by exploring students' behavior and interaction patterns in different types of online quiz-based activities within learning management systems (LMS) (Juhaňák et al., 2019). Especially, many studies of SPP were applied to discover a better learning pattern and thereby improve the educational output (Hu and Huang, 2018; Lam et al., 2018; Kushwaha et al., 2019; Supianto et al., 2019).

## 3. Comparison Experiments

This section adopted traditional machine learning algorithms to predict student performance on two data sets, i.e., a private data set from our institution and a public data set.

### 3.1. Data Description

One of the two data sets was collected during the 2005 and 2006 academic years from two Portuguese schools (Dataset 1). Data features include student grades (the grades of the three semesters are labeled as G1, G2, and G3), demographic features, social features, and school-related features. The data set provided two distinct courses, *Mathematics* and *Portuguese Language*. This data set can be obtained from https://archive.ics.uci.edu/ml/datasets/Student+Performance.

The other data set was collected from the Computer Science department at our institution on 694 undergraduate students of 2014, 2015, and 2016 (referred to as Dataset 2). The data contains student background features (e.g., Gender, class, age, nationality, political status), course credits, course hours of one week, and grades on 39 courses. The 39 courses were taken in different semesters.

### 3.2. Problem Reformulation

We recast this student grade prediction into classification and regression.

- Classification formulation: Dataset 1 was classified based on the Erasmus grade conversion system, where there were 5 Levels. While Dataset 2 was classified based on student evaluation with 3 Level grades. The level details of the two data sets are shown in Table 5.
- Regression formulation: Dataset 1 has numeric outputs ranging from 0 to 20, and Dataset 2 has numeric outputs varying from 0 to 100.

**Table 5:** The details of the two datasets used in experiments.

Dataset 1	0-9	10-11	12-13	14-15	16-20
	fail	sufficient	satisfactory	Good	Excellent
Dataset 2	grade <60	60 ≤ grade ≤ 80	80 < grade		
	Warning	Good	Very Good		

### 3.3. The Used Methods

In this study, the aim is to summarize current works and have comparisons between the used methods. Hence, we employed those methods that had been adopted in related references, as follows:

**Naive Bayes**: Naive Bayes is based on Bayes' theorem with feature condition independent hypothesis. For the training set, the joint probability distribution of input and output is first studied based on the independent hypothesis of feature conditions. Then based on this model, Bayes theorem is used to calculate the *y* with the maximum posterior probability for the given input *x*.

k-nearest neighbor is a basic classification and regression method in machine learning. KNN was used by Cover and Haut for SPP (Cover and Hart, 1967). KNN first determines the on the K training data set for a test data point, and then use the majority of the classes of the *k* training data points to predict the classes of the test point.

**Decision tree**: A decision tree is a commonly used classification and regression method in SPP. The algorithm consists of three parts, i.e., feature selection, tree generation, and pruning. The main implementation includes ID3 and C4.5 proposed by Quinlan (1986), and CART proposed by Breiman et al. (Loh, 2011). In this experiment, we used the C4.5 algorithm.

**Support vector machine**: Vapnik originally proposed SVM, and Chervonenkis (Vapnik and Chervonenkis, 1964; Cortes and Vapnik, 1995). Boser *et al*. proposed a non-linear SVM by using kernel methods and soft margin maximization (Boser et al., 1992). Weston et al. extended it to multi-classification (Weston and Watkins, 1999). We here used the sequential minimal optimization (SMO) algorithm proposed by Platt (1998).

**Bagging**: Breiman proposed bagging (Breiman, 1996). Bagging is a technique that reduces generalization errors by combining several models. The main idea is to train several different models separately and then let all models vote for the output. In this experiment, we adopted the C4.5 algorithm as the base classifier.

**Random Forest**: Random Forest is an ensemble learning method proposed by Breiman (2001). It uses voting mechanisms from multiple DT to improve the shortcomings of DT. In this experiment, we chose the C4.5 algorithm as the base classifier, a.k.a. weak classifier.

### 3.4. Model Training and Parameter Selection

We implemented all methods by using Weka3.8 ^1^ software. All experiments were evaluated with 10-folds cross-validation (Garćıa et al., 2010). That is, we partitioned the dataset into 10-folds and performed the evaluation ten times. In each evaluation, 1-fold was used as the test set, and other folds were used as the training set. After ten runs, the average metrics were calculated as the final evaluation results. In our classification experiment, models were evaluated using the prediction accuracy (ACC), while the root mean squared error (RMSE) in the regression experiment. Two-sample t-test was adapted to verify the statistical significance of the difference between the two methods (Sheskin, 2003).

Hyperparameter selection is an important task to extract more accurate results. Grid Search is generally used for hyper parameter optimization. In grid search, different models having different parameter values are trained and then evaluated using cross-validation. The 10-folds cross-validation could be performed on each model, and the hyper parameters with optimum results are then selected. For Naive Bayes, KNN, DT, SVM, Bagging and Random Forest, one could used grid search 10-folds cross-validation for hyper parameters selection for each model, and then perform model training and comparison with the selected parameters. The used hyperparameters are: *K* = 5 for KNN; *c* = 2 for SVM; the number of features = 6 for Random Forest; *n* = 100 for bagging and boosting. The other parameters that are not mentioned here are set to default values in Weka.

### 3.5. Experiments

We conducted experiments on two education data sets to predict student grades on specific courses to compare these mentioned methods. We additionally investigated feature effects in SPP.

#### 3.5.1. Effects of Previous Courses

As many researchers mentioned, a student's grades are closely related to his previous grades. In the prediction experiments of *Portuguese* and *Mathematics* grades in Dataset 1, the features G1 and G2 are thought to have great influences on G3. Therefore, each DM model has three input configurations:

A: Use background features (demographic features, social features, and school features) to predict G3.
B: Add G2 based on A.
C: Add G1 based on B.

**Figure 1:**
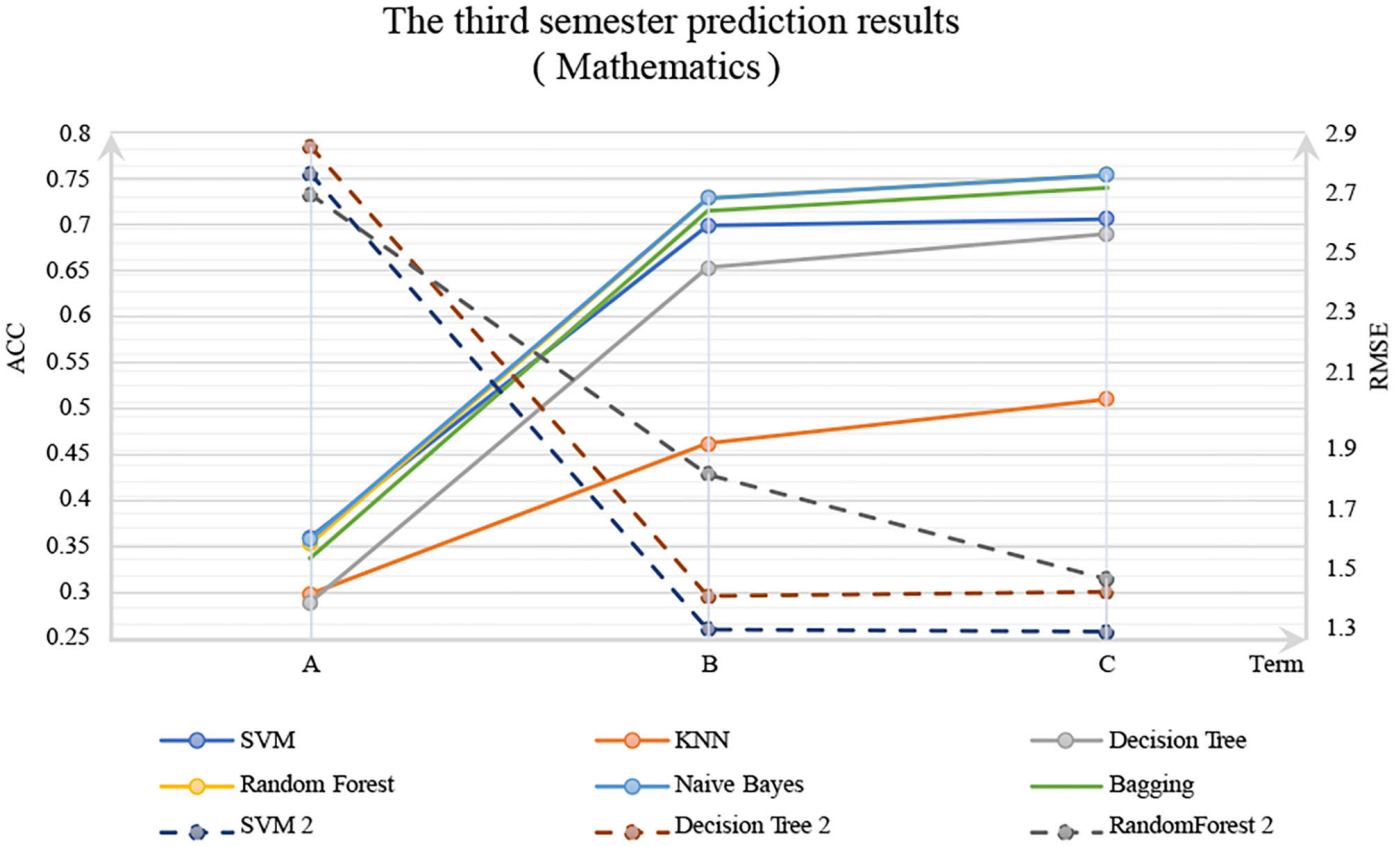
Mathematics.

**Figure 2:**
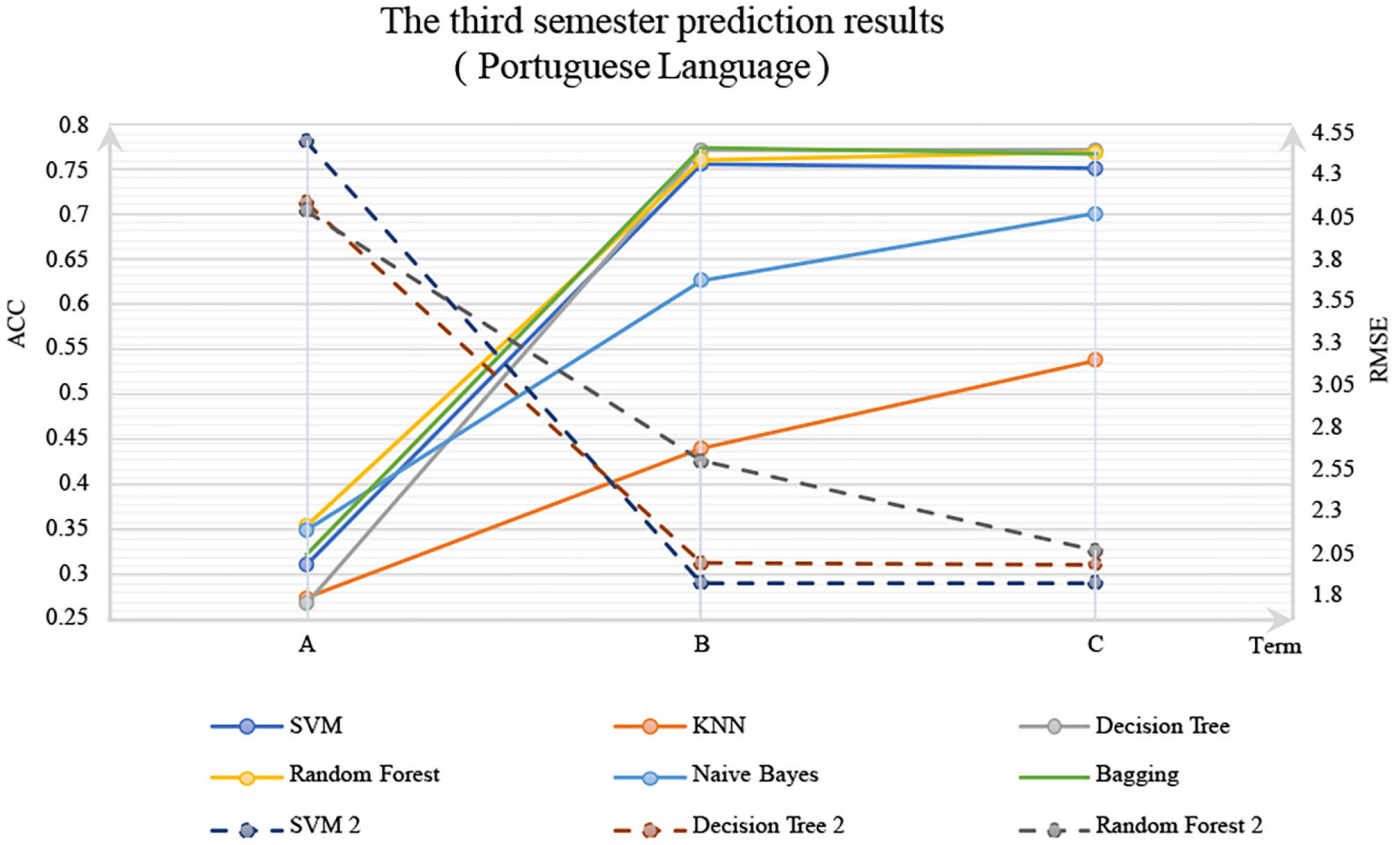
Portuguese language.

The experimental results are shown in Figures 1, 2. In these figures, the solid line is the classification method, ACC is marked on the left primary axis as the evaluation metric, and the dotted line is the regression method, and RMSE is marked on the right secondary axis as the evaluation metric. As shown in Figure 2, when only background features are used to predict G3, poor prediction results are delivered. The ACC of DM models is about 0.3, and RMSE is about 0.75, indicating that background features have little influence on G3. However, when G2 and G1 were considered, the accuracy was increased significantly with a *p*-value <0.05, and RMSE decreased significantly, indicating that the performance of the DM model could be improved by adding the grades of prerequisite courses. In particular, with G2, Random Forest reaches 0.75 on classification accuracy, and SVM for regression reaches about 0.25 on RSME. The similar trend could be observed in Figure 2 as well.

#### 3.5.2. Grade Prediction on Specific Courses

In Dataset 2, we selected a course grade in the sixth semester (*Operating System*) and a course grade in the fifth semester (*Computer Composition and System Structure*) to survey these mentioned methods. All models were set to different input configurations corresponding to the different number of semesters and the different number of prerequisite courses. Here, we took the sixth semester as an example (The fifth semester is similar to the sixth semester):

A: Using background features and other course features from the same period of the sixth semester.
B: Adding the course features of the fifth semester to settings A.
C: Adding the course features of the fourth semester to settings B.
D: Adding the course features of the third semester to settings C.
E: Adding the course features of the second semester to settings D.
F: Adding the course features of the first semester to settings E.

**Figure 3:**
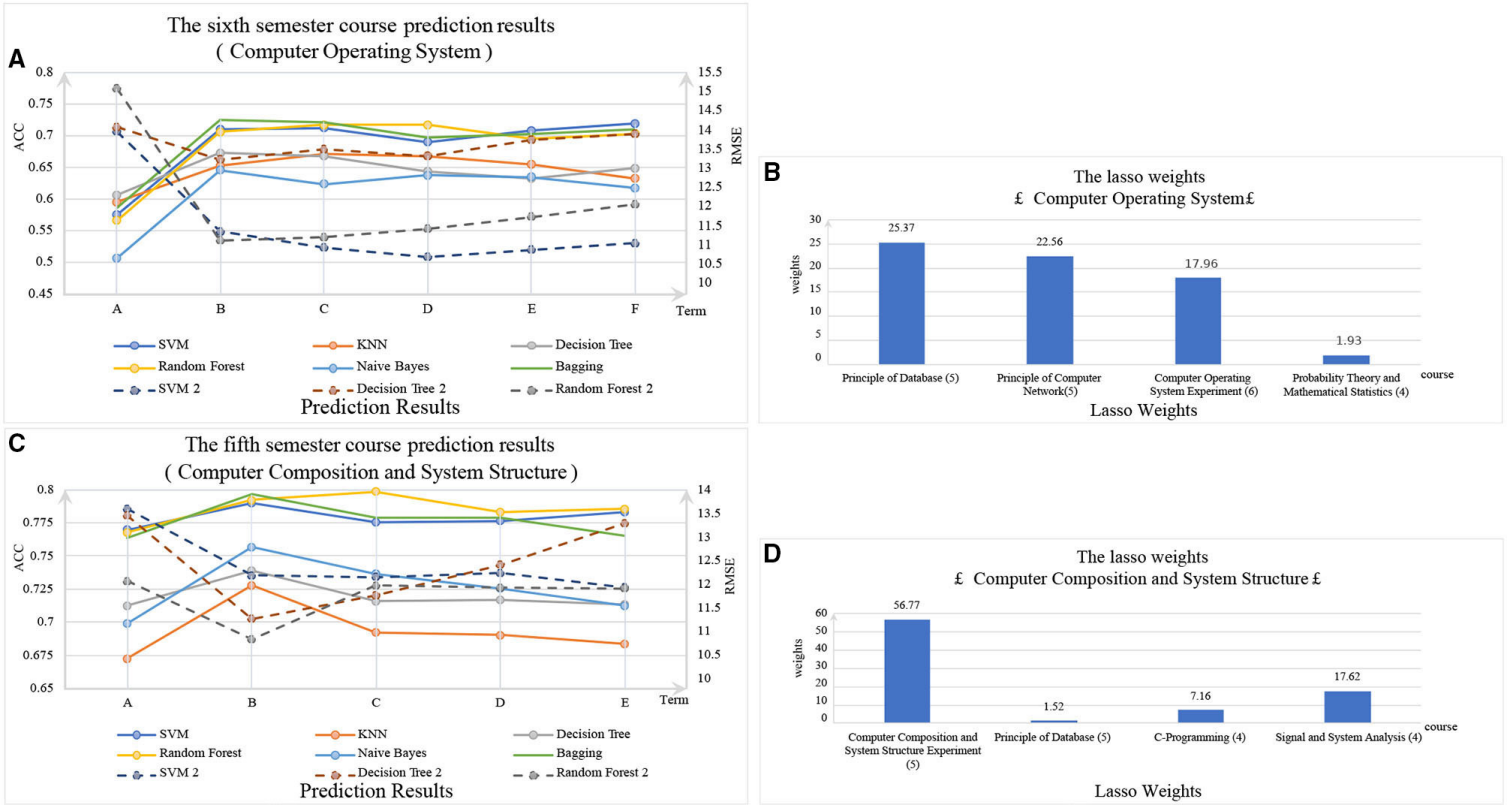
In **(A,C)**: The solid lines are classification methods, and ACCs are marked on the left primary axis as the evaluation metric. The dotted lines are regression methods, and RMSEs are marked on the right secondary axis as the evaluation metric. In **(B,D)**: The x-axis is the course whose weights are not zero after the feature selection by Lasso, and (*) represents the semester of the course. The y-axis is the weight of the course.

The experimental results are shown in Figures 3A,C. It can be seen that with the increase of the number of prerequisite courses, ACC increases and RMSE decreases. The performance of all models could be improved by increasing the grades of prerequisite courses. However, although the ACC is increased, it is not monotonously increasing. However, RMSE is decreased, but not monotonically decreasing. More noise may be introduced as the number of semesters increases. Thus, we used the Lasso algorithm for feature selection. The Lasso algorithm gives the weights of these features in the prediction process by compressing the weight of the uncorrelated and redundant features to zero. Figures 3B,D, respectively, show the weights of relevant courses. As can be seen, the last semester and the same semester of course account for greater weight. The weights of background features and other semester course features are zeros. Therefore, in Dataset 2, background features have less influence on the prediction of grades, while some courses in the last semester have greater influences on the predicted grades.

### 3.6. Result Analysis and Conclusion

From the experiment results above, the most accurate classifier is Random forest. Random forest is an ensemble learning algorithm by combining multiple weak classifiers and the final results are obtained by voting or averaging those multiple weak results, resulting in high accuracy and better generalization performance. Wherein, the prerequisite course grades play the most important role in the random forest classifier, which shows that the records might uncover the characters of a student on learning. In addition, the performance of all models is significantly improved with integrating the features from the prerequisite courses (*p*-value <0.01). On the other hand, the background features have a small influence on the predictions. Overall, if we could access more information on the prerequisite courses, the performance of all models would be better.

Specifically, after feature extraction on Dataset 2, the courses of last semester and the courses in the same term make a great influence on the grade prediction. In contrast, other features have small influences, while these redundant features might introduce noise. In the task of SPP, it is thus necessary to select informative features and remove redundant features to reach a better performance. By the way, these study results could convince the conclusions in the work of Debopam et al. (Sanyal et al., 2020).

## 4. Ethical Considerations on SPP Algorithms

There are lots of successful algorithms to predict the student grade mentioned above. While some institutions use the SPP algorithm to guide the students on learning contexts and/or learning pathways, the ethic should be considered for the real-world use of a computer algorithm due to the agnostic of the impacts on education.

### 4.1. Data Access and Collection

This study motivates us to continuously collect more educational data from real-world education, while privacy issues arose to be taken into account (Ekowo and Palmer, 2017). The educational data consists of many important personal information which could be harmful if the data is leaked.

Morozov et. al. proposed three provocative positions in this shift toward algorithms (Morozov and Morozov, 2013). That is, (1) personal privacy needs to be politicized in data intensive problem solving under scrutiny; (2) the value of the personal data needs to be considered as a shift thinking; (3) data sharing needs to be carefully conducted in the development of provocative digital services. Princloo et al. extended into five theses to make sure the secure use of data (Prinsloo et al., 2015), which were focused on data access, the proposition of an integrated data system, the skills and capability to manage data, and systematically mapping the data elements for reporting and analytics, respectively.

These suggestions were concluded into three points by Angelo et al. (Fynn, 2016), i.e., data access, the value of personal data, and the data origins. For data access, he advocated a data analytics framework should be set up to consider the institutional assumption, practices, and ideology underpinning the data mining technology. For the value of personal data, it should be clearly claimed that student remains the option to save or remove data and the predictive analysis has positive effects on students at high risk. For data origins, student success is a complex phenomenon from different predictor variables across institution types, material resources, heterogeneous student, socio-economic status, disciplinary contexts, and so on.

### 4.2. Algorithm Threat and Advantage

Researchers could access a huge amount of educational data, and they considered the artificial intelligence algorithms to enhance the performance of SPP tasks, i.e., KNNs, SVMs, MFs, etc. Students and teachers enjoy the advantage of algorithms, while suffering from their threats (Ekowo and Palmer, 2016; Fynn, 2016).

These algorithms reviewed above improved the performance of SPP, finding students at-risk, and further bring out targeted student advising. These advantages enhances the educational quality, reduce the drop-out ratios, and help students be better. On the other hand, the education might suffer from these algorithms, leading to agnosticism. The threat lies in 2-folds: the invisible control of our behaviors to meet an undisclosed ideological agenda and the unknown decision making from an agnostic algorithm (Fynn, 2016). Most models focus on the improvement of their used metrics, while less consider the interpretability. The use of these algorithms put us in an unsafe situation.

Several types of research start to pay attention to the reason hiden in algorithms. Zhang et al. proposed one robust MF model to solve the SPP task, while integrating a graph to improve the interpretability (Zhang et al., 2020c). In the recent works (Zhang et al., 2021a), the authors attempt to probe the reason why the target students will fail on a course by using the course relationship. The predicted grade without any interpretation will not be convinced and could not apply in a real educational environment. All in all, we should be reasonable about the benefits and disadvantages and develop a more useful educational analysis model with strong interpretability.

### 4.3. Analytic Bias

The study on students often is effected by race, gender, age, finance, and so on, leading to model bias and unfairness in practices. As mentioned in the study of Jiang et al. (Ekowo and Palmer, 2016), education fairness and algorithm fairness are important to the SPP task. Jiang et al. hold the point that analytic bias consists of educational data bias and algorithm bias. In the research (Ekowo and Palmer, 2016), they introduced the SPP task into three stages: data construction, model training, and inference. Then, Jiang designed some strategies for the three stages, i.e., weight loss by sample strategy for data constracution, adversarial learning for model training, and removing features for prediction in the last stage. Mostly, the scores could not truly reflect students' ability level, where two students with similar reviews may obtain different score on open-ended assignments (Cleary, 1968). Besides, Jordan et al. studied the race problem to show the reasons why institutions of higher education may choose to embrace diversity (Starck et al., 2021); Natalia et al. studied the gender gap in STEM to show the difference of mathematics anxiety between boys and girls (Ayuso et al., 2020).

## 5. Discussions and Future Works

**Figure 4:**
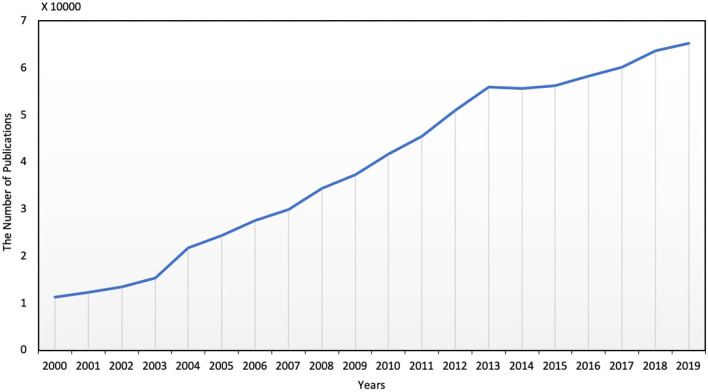
The number of publications for student performance prediction per year.

Although the increasing works have been devoted to SPP, shown in Figure 4, many limitations and large improvement spaces still exist. In this review, we listed some key concerns but not limited to them.

### 5.1. Open Data Requirement

This review shows that the current works only focus on their private data sets, while a few data sets could be publicly available at http://educationaldatamining.org/resources/. Unavailable data sets cause three issues. First, the focuses are different among different situations, so that researchers lose the focused scientific point that is needed in learning analytics and EDM. Second, fair comparisons can not be conducted due to the different data sets used in these reviewed papers. Finally, the data problem hinders these proposed models from valuations and applications. Toward this problem, more data sets by removing private information might be created and opened to use for this research field in the future.

### 5.2. New Methods With Education Priors

Student performance prediction problems involved data mining techniques, psychology, educational theories, etc. Current studies mostly focus on the uses of traditional machine learning methods, ignoring the prior knowledge from educational practices. The priors could be (1) the course organization in school or university (Zhang et al., 2020c), which affects the objective courses by the knowledge association between courses. (2) the learning curve of students (Gallistel et al., 2004), which shows the different weights on the objective course predictions. (3) the prerequisites of the objective course (Chen et al., 2016), which plays an important role in the objective course prediction. In addition, the side-information features, like learning behaviors in the free time and the learning attitudes, significantly affect the learning performance on courses. On the other hand, SPP should be concerned with learning science rules, which could help achieve more precise performance and interpretable results.

Besides, there are many complicated factors for course grades like family, campus life statistics,s and learning psychology, which have been proved by the educators that affect student academic performance (Li and Zaman, 2014). This helps to improve the model accuracy but does not provide a stronger directional educational conclusion. In future work, the SPP method could consider the combination of EDM techniques and educational theories and priors.

### 5.3. Result Interpretation for Education

The current works paid more attention to the prediction accuracy of course learning, using various models, including deep learning models. The result explanations in learning and teaching are few but more important. Yupei et al. explained the student grade prediction by discovering the relationships between courses (Zhang et al., 2021a). The prediction could guide course selection and early warning on student learning, but finding the key factors affecting most education behaviors is a more important task in SPP. That is because (1) the key feature could correspond to interventions of education; (2) the reason of success or failure could reflect the pattern of student learning; (3) understanding of these factors could provide plan settings, course assignments, and learning sequence with suggestions.

For interpretation, the traditional methods, such as LR and DT, are more promising than a complex method. While the learning process is hard to understand now, the deep model could have more effectively fit the data to learn the pattern (Kim et al., 2018; Su et al., 2018; Zhang et al., 2021a). Hence, the trade-off between the accuracy and model complexity is considered for the SPP model in practice. In the future, integrating more education priors into prediction to enhance the explanation of the results of SPP is a significant topic. Feature engineering, using manual features, feature selection, or explainable features, is also an open problem in future works.

### 5.4. Personalized Education System

Personalized service is important on the Internet, especially web-based learning. A personalized education system is a supplementary learning tool within the traditional study that could provide high-efficient learning guidance. For the further development of SPP, building an educational personalized expert system is one way to put the research conclusion into practice. With the technique of SPP, students will receive help during their study process. This system will give a good learning path to students. Recent researches on personalized system consider some plain features such as learner preferences, interests, and browsing behaviors (Liaw, 2008). To improve the accuracy of SPP, we need to put more latent features into the system. Then, designing software to integrate the SPP is another important work in computer-aided teaching and learning. However, integrating these models into the expert system and using the conclusion guide the teaching procedure is still needed to explore and probe.

## 6. Conclusion

As it is an important evaluation of educational outputs, student performance plays an important role in EDM research. Moreover, predicting student performance could help learners and educators improve their learning and teaching. However, the current studies are limited in statistical methods or educational theory, while it does not attract attention to using the popular techniques, i.e., feature learning (Zhang et al., 2020b). Besides, the existing studies are lacking fair comparisons between various developed methods with fair metrics and fair validated datasets.

This paper reviews previous studies from the five data mining steps, including data collection, problem formulation, used method, prediction target, and practical applications. Specifically, we reviewed three education styles, i.e., online, offline, and blending courses. As the online classroom increases, big data is easy to collect to enhance the importance of SPP. Then, we went through these studies and partitioned them into different groups to have a research summary. Besides, we conducted evaluation experiments on the two data sets from different situations to compare the involved methods, including a private data set from our institution and a public data set. The result shows that (1) the method-learning methods could achieve a good performance on SPP, and (2) feature selection could boost SPP. The evaluation also delivers a suggestion on the importance of pre-required courses. In addition, on our data sets, the relationships between those chosen attributions, e.g., *Principle* *of Database* is more related with *Computer Operating System* than *Principle of* *Computer Network*. Thus, for better prediction results, we could choose the suitable features that are mostly related to student performance through feature selection, e.g., Lasso and its variances (Zhang et al., 2018b, 2021b). After reviews and the case study, we discussed the issues and advantages of current works and future studies.

On the one hand, this comprehensive review on this young inter-discipline from machine learning and data mining motivates us to develop more popular methods for SPP. On the other hand, future studies should consider the priors from the education field to develop domain-specific machine-learning models. This research could help the education system pursuit better educational outcomes while reducing both educational finance and cost. In the future, we will develop more educational machine-learning methods for SPP and other studies in EDM (Zhang et al., 2020a).

## Data Availability Statement

The raw data supporting the conclusions of this article will be made available by the authors, without undue reservation.

## Ethics Statement

The studies involving human participants were reviewed and approved by School of Computer Science, Northwestern Polytechnical University. Written informed consent for participation was not required for this study in accordance with the national legislation and the institutional requirements.

## Author Contributions

YZ designed the paper and experiments, wrote this paper, and funded this study. YY wrote the draft of a review on SPP process and ethical considerations on SPP algorithms. RA conducted the experiments and wrote the draft of comparison experiments. HD and JC wrote the draft of discussions and future works. XS supervised and funded this study. All authors contributed to the article and approved the submitted version.

## Funding

This study was supported in part by National Natural Science Foundation of China (Grant Nos. 61802313, U1811262, and 61772426), Key Research and Development Program of China (No. 2020AAA0108500), and Reformation Research on Education and Teaching of Northwestern Polytechnical University (No. 2021JGY31).

## Conflict of Interest

The authors declare that the research was conducted in the absence of any commercial or financial relationships that could be construed as a potential conflict of interest.

## Publisher's Note

All claims expressed in this article are solely those of the authors and do not necessarily represent those of their affiliated organizations, or those of the publisher, the editors and the reviewers. Any product that may be evaluated in this article, or claim that may be made by its manufacturer, is not guaranteed or endorsed by the publisher.

## Footnotes

^1^ https://www.cs.waikato.ac.nz/ml/weka
