# Corrigendum: Educational Data Mining Techniques for Student Performance Prediction: Method Review and Comparison Analysis

**DOI:** 10.3389/fpsyg.2021.842357

**Published:** 2022-01-21

**Authors:** Yupei Zhang, Yue Yun, Rui An, Jiaqi Cui, Huan Dai, Xuequn Shang

**Affiliations:** ^1^School of Computer Science, Northwestern Polytechnical University, Xi'an, China; ^2^Key Laboratory of Big Data Storage and Management, Ministry of Industry and Information Technology, Xi'an, China

**Keywords:** personalized education, review and discussion, educational data mining (EDM), student performance prediction, pattern recognition

An author name was incorrectly spelled as **Xunqun Shang**. The correct spelling is **Xuequn Shang**.

The authors apologize for this error and state that this does not change the scientific conclusions of the article in any way. The original article has been updated.

## Publisher's Note

All claims expressed in this article are solely those of the authors and do not necessarily represent those of their affiliated organizations, or those of the publisher, the editors and the reviewers. Any product that may be evaluated in this article, or claim that may be made by its manufacturer, is not guaranteed or endorsed by the publisher.

